# Through Another’s Eyes: Implicit SNARC-like Attention Bias Reveals Allocentric Mapping of Numerical Magnitude

**DOI:** 10.3390/bs15081114

**Published:** 2025-08-17

**Authors:** Wanying Luo

**Affiliations:** School of Psychological and Cognitive Sciences, Peking University, Beijing 100871, China; wanying@pku.edu.cn

**Keywords:** numerical–spatial mapping, spatial attention, embodied perspective taking, allocentric reference frame, implicit social cognition

## Abstract

Numerical magnitude can bias spatial attention, typically facilitating faster responses to the left for small numbers and to the right for large numbers—an effect traditionally attributed to egocentric spatial mappings. However, in everyday environments, individuals often share space with others, raising the question of whether such spatial–numerical associations can spontaneously reorganize based on another person’s visual perspective. To investigate this, we employed a digit-primed visual detection paradigm in which participants judged the location (left, right, up, or down) of a briefly presented peripheral probe following centrally displayed digits. If numerical magnitude implicitly guides attention, probe detection should be faster when its location is congruent with the digit-induced spatial bias. Critically, in the avatar condition, a task-irrelevant avatar was positioned on the participant’s left side, such that the avatar’s horizontal (left–right) axis corresponded to the participant’s vertical (up–down) axis—an axis along which egocentric numerical biases are typically absent. If participants spontaneously adopted the avatar’s perspective, numerical cues might induce attentional biases along this axis. Results revealed two simultaneous effects: a canonical egocentric SNARC-like effect (small–left, large–right) and a novel allocentric effect (small–up, large–down) emerged along the vertical axis, implicitly aligned with the avatar’s left–right spatial orientation. Numerical extremity enhanced the egocentric SNARC-like effect but had no effect in the allocentric case, pointing to a distinct mechanism rooted in embodied spatial perspective. These findings suggest that numerical magnitude can implicitly map onto both egocentric and allocentric spatial frames, reflecting a implicit and embodied mechanism of social understanding.

## 1. Introduction

Responses to numerical stimuli are systematically biased along spatial dimensions, with smaller numbers typically associated with the left and larger numbers with the right—an effect known as the Spatial–Numerical Association of Response Codes (SNARC; Gevers & Lammertyn, 2005; Tzelgov et al., 2013; Winter et al., 2015; Rugani et al., 2015). This phenomenon has been widely interpreted as evidence for a “mental number line”, a culturally shaped spatial representation that maps numerical magnitude onto horizontal space (Fias & Fischer, 2005). Critically, this mapping has predominantly been studied within an egocentric reference frame, where spatial associations are anchored to the observer’s left–right bodily axis.

However, in daily life, numerical information—prices on a menu, scores in a sports game, or classroom counts—is rarely processed in isolation (Saxe et al., 1987; Atmaca et al., 2008). Instead, it is often embedded within complex social environments that include other people, whose physical positions and spatial perspectives differ from one’s own. This raises a fundamental and underexplored question: Are spatial–numerical associations rigidly constrained by the observer’s egocentric frame, or can they flexibly reorganize based on another person’s point of view, even when that viewpoint is incidental or task-irrelevant?

This question resonates with research on embodied visual perspective taking (VPT), which reveals that individuals can spontaneously simulate others’ visuospatial experiences. Even in the absence of explicit instruction, people often mentally adopt another person’s spatial viewpoint by projecting themselves into the other’s position and imagining the world from that perspective (Tversky & Hard, 2009; Samson et al., 2010; Kessler & Thomson, 2010). Such embodied simulations are sensitive to spatial parameters like orientation, distance, and bodily alignment, and they have been shown to influence attention, spatial coding, and motor responses (Michelon & Zacks, 2006; Ward et al., 2019, 2020, 2022). For example, Kessler and Rutherford (2010) found that participants were better at perspective-taking tasks when their posture matched that of an avatar, suggesting that sensorimotor alignment facilitates adopting another person’s viewpoint. Furthermore, Ward et al. (2019) demonstrated that embodied simulation leads to vivid perceptual experiences, as if the observer is literally “seeing” through another’s eyes. Although the majority of studies on VPT have focused on concrete visual features or object properties (e.g., determining where and what another person can see), they raise the intriguing hypothesis that even abstract spatial associations—such as those linking numbers to horizontal space in the SNARC effect—might likewise be modulated through embodied perspective taking.

More recent evidence extends this flexibility into socially embedded contexts. Müsseler et al. (2019) reported that SNARC effects could reverse when a front-facing avatar appeared opposite the participant, and that participants could actively suppress or allow such perspective-based reversals depending on task demands—suggesting a controllable component in adopting allocentric frames. D’Ascenzo et al. (2022) further demonstrated that number–space compatibility effects emerged only in human–human joint go/no-go tasks, but not in non-social versions, highlighting the critical role of sociality in driving spatial–numerical reorganization.

Crucially, some studies suggest that perspective-related modulations of spatial–numerical associations may occur even without explicit perspective-taking instructions. For example, Böffel et al. (2021) found that the mere presence of a laterally positioned avatar could elicit a SNARC-like effect aligned with the avatar’s spatial axis. This has been interpreted as evidence for covert motor alignment, whereby participants implicitly remap their response tendencies to match the avatar’s reference frame. However, such interpretations primarily focus on action-based processes (Yan et al., 2021) and leave open the question of whether earlier, attention-related spatial codings can also be shaped by another person’s presence. Specifically, can attentional orienting itself—prior to any motor preparation—be implicitly biased by another agent’s spatial perspective?

This distinction is critical. If spatial biases in attention can be reorganized according to another person’s reference frame, it would provide strong evidence that number–space mappings are not only motorically flexible but also perceptually reconfigurable in a social context. This would support the idea that embodied simulations of others’ spatial perspectives can influence not just late-stage response selection but early-stage perceptual prioritization and attentional allocation.

To directly test this possibility, the present study investigates whether a task-irrelevant social agent can implicitly shift an attentional allocation originally biased by an egocentric reference frame. We adopted a digit-primed visual detection paradigm: centrally presented numbers were used to trigger lateral shifts of attention consistent with the standard SNARC effect, followed by a peripheral visual probe requiring rapid detection. In the avatar condition, a front-facing avatar was positioned to the participant’ s left, serving as a salient yet entirely task-irrelevant external reference frame.

Importantly, participants were never instructed to adopt the avatar’s perspective. Thus, any modulation of spatial attentional effects—such as enhanced detection when the probe aligns with the avatar’s left or right—would not reflect deliberate perspective taking or strategic motor alignment but rather an implicit remapping of the original egocentric spatial code into the allocentric frame. In the present study, the term “allocentric” is used to refer to a social reference frame centered on another agent’s body and viewpoint. This is distinct from the traditional, map-based allocentric coding in the spatial navigation literature (e.g., C. Wang et al., 2020; Ekstrom & Isham, 2017). Rather, the usage aligns with embodied accounts of visual perspective taking, wherein individuals implicitly simulate another’s spatial viewpoint (Kessler & Rutherford, 2010; Ward et al., 2019). Accordingly, attentional shifts initially driven by numerical magnitude in an egocentric reference frame may be spontaneously reorganized in alignment with the spatial axis of a socially present agent.

Moreover, we examined whether such effects are modulated by numerical extremity—that is, whether more extreme digits (1/9) elicit stronger spatial biases than mid-range ones (3/7). This analysis probes whether magnitude sensitivity extends into allocentric mappings and whether numerical salience strengthens embodied remapping (Leibovich et al., 2017).

In sum, the present study advances the literature in three key ways. First, it investigates whether abstract numerical–spatial associations can be implicitly remapped into an allocentric frame based solely on social presence. Second, it targets early attentional processes, extending prior work that has focused predominantly on motor responses. Third, it offers a novel paradigm for studying how numerical cognition interacts with embodied social perception, shedding light on the interplay between abstract symbol processing and real-world social understanding. In this way, the present study provides a novel illustration of how conceptual number–space associations are dynamically shaped by social context, underscoring the profound interplay between abstract conceptual processing and embodied social cognition.

## 2. Materials and Methods

### 2.1. Participants

We conducted a power analysis using G*Power 3.1.9.7 to determine the required sample size. We assumed a 0.95 power, a large effect size of 0.40, and a 0.05 alpha error probability; thus the program yielded a sample size of 24. Additional data was collected to ensure that any exclusion of data as a result of extreme scores or non-compliance of the task would not cause a reduced cohort. Ultimately, this study comprised 26 participants (15 female; mean age = 21.769 years, range = 18–26, SD = 2.065). All were right-handed, had normal or corrected-to-normal vision, and reported no history of neurological or psychiatric disorders. Informed consent was obtained from all participants prior to the experiment. The procedure was approved by the ethics committee of Peking University and complied with the Declaration of Helsinki.

### 2.2. Materials and Stimuli

The numerical stimuli consisted of Arabic digits from 1 to 9, excluding 5. Digits 1–4 were classified as “small” and 6–9 as “large”. The detection stimulus was a white solid dot presented on a black background. All stimuli were displayed on a 27-inch LCD monitor (refresh rate: 60 Hz; resolution: 1920 × 1080), with participants seated approximately 60 cm from the screen.

Numerical stimuli were presented centrally in a white font, approximately 1.5° of visual angle in height, and remained onscreen for 500 ms. After a 200 ms blank interval, the detection probe appeared at one of four equidistant peripheral locations (up, down, left, right), each approximately 6° from the screen’s center. The probe remained onscreen until a response was made.

In the avatar condition, a seated avatar image (up–down view, facing forward) was fixed on the left side of the screen, measuring approximately 6° × 8°. It remained consistently positioned in the left visual field and provided a salient yet task-irrelevant allocentric frame of reference. The avatar appeared immediately after the digit and stayed on screen until the response. In the baseline condition, no avatar image was presented.

### 2.3. Design and Procedure

**Figure 1:**
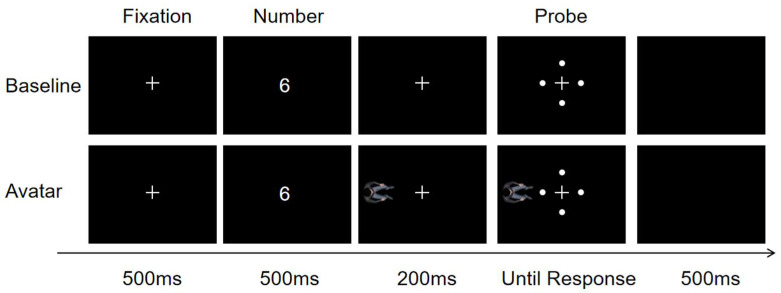
Schematic depiction of the trial procedure in the baseline and avatar conditions. In each trial, a centrally presented digit (1–4 or 6–9) was shown for 500 ms, followed by a 200 ms interstimulus interval (ISI). A small white dot (the probe) then appeared in one of four peripheral locations (left, right, up, or down) and remained onscreen until the response. Participants were instructed to detect the dot’s location as quickly and accurately as possible. In the baseline condition, no additional stimuli were shown. In the avatar condition, a task-irrelevant avatar was consistently presented on the participant’s left side, facing the center. The avatar’s left–right axis corresponded to the participant’s vertical (up–down) axis, enabling measurement of potential allocentric spatial–numerical congruency effects.

The experiment followed a 2 (social condition: avatar/baseline) × 2 (numerical magnitude: small/large) × 4 (probe location: up/down/left/right) within-subject design. Each condition included 40 trials, totaling 640 experimental trials, with each specific digit presented 10 times per condition. An additional 32 catch trials were included (~4.76% of all trials). These trials followed the same temporal sequence as regular trials but ended with a multiple-choice prompt requiring participants to report which digit had just been presented, ensuring attention to the numerical stimuli (see Figure 1 for a visualization of the experimental procedure).

Trials were randomized. Each trial began with a 500 ms central fixation cross, followed by the digit, blank interval, and detection probe. Participants were instructed to respond as quickly as possible to the location of the probe using the numeric keypad: up = 8, down = 2, left = 4, right = 6. Responses were made using the right index finger, which returned to the center (key 5) after each response. Gaze was to be maintained centrally throughout.

### 2.4. Data Recording

Accuracy and reaction times (RTs) were recorded for each trial. Only correct trials were included in the RT analysis. Trials with RTs below 100 ms or above 1000 ms were excluded as outliers. The primary dependent measure was the mean RT for each condition per participant.

### 2.5. Data Analysis

We first evaluated participants’ performance on catch trials to ensure attentiveness to the centrally presented digit primes. All participants passed this attention check, making fewer than three errors across 32 catch trials. The average accuracy was 98.68%, indicating consistent engagement with the digit stimuli throughout the experiment.

Prior to analyzing spatial–numerical effects, we confirmed that no systematic response bias was associated with key mappings. Mean reaction times (RTs) for left and right responses in baseline trials were compared and showed no significant difference, ruling out low-level motor asymmetries as a confound.

To assess how numerical magnitude mapped onto spatial probe locations across different reference frames, we defined four trial types by crossing number size (small vs. large) with probe location (left–right in the baseline condition, up–down in the avatar condition):

In the baseline condition, “self-consistent” trials were those in which small numbers were followed by left-side probes and large numbers by right-side probes, in line with the classic horizontal SNARC effect. “Self-inconsistent” trials violated this mapping, pairing small numbers with right-side probes and large numbers with left-side probes.

In the avatar condition, “avatar-consistent” trials were defined relative to the avatar’s vertical axis, with small numbers followed by up-side probes and large numbers by down-side probes—consistent with a vertical SNARC-like mapping from the avatar’s viewpoint. “Avatar-inconsistent” trials reversed this mapping.

These trial classifications, summarized in Table 1, enabled direct comparisons between consistent and inconsistent pairings of numerical magnitude and spatial probe location, allowing us to assess whether SNARC-like effects emerged along either egocentric or allocentric axes and to quantify their relative strength.

To examine whether numerical extremity modulated the strength of spatial–numerical associations, we computed difference scores separately for small and large numbers, based on the direction of expected spatial associations in each reference frame. In the egocentric condition, for small numbers (1–4), we subtracted reaction times for left-side probes from those for right-side probes (i.e., right minus left), because small numbers are typically associated with leftward responses. For large numbers (6–9), we subtracted right-side probe RTs from left-side probe RTs (i.e., left minus right), as large numbers are typically associated with rightward responses. In the allocentric condition, we applied a similar logic using vertical probe positions. For small numbers, we subtracted RTs for lower probes from those for upper probes (up minus down), while for large numbers, we subtracted upper-probe RTs from lower-probe RTs (down minus up), consistent with the avatar-defined spatial mapping. We then compared these scores between central digits (3/4 and 6/7) and extreme digits (1/2 and 8/9), to test whether greater numerical distance from the midpoint enhanced spatial–numerical congruency effects.

Given the low overall error rates, accuracy was not analyzed further. Mean error counts per participant were minimal across probe positions—left = 0.731, right = 0.423, up = 1.077, and down = 1.192—suggesting that the task was well understood and consistently performed.

## 3. Results

### 3.1. Preliminary Check: Response Key Bias

To rule out low-level motor or response mapping confounds, we first assessed whether the four response keys differed in baseline reaction times (RTs). Paired-sample *t*-tests were conducted across all non-catch trials to compare mean RTs between left and right keys, as well as between up and down keys. Results revealed no significant difference between left and right responses, *t*(25) = 1.553, *p* = 0.130, nor between up and down responses, *t*(25) = −1.564, *p* = 0.133. These findings suggest that the observed effects in later analyses are unlikely to be attributable to systematic motor biases or key-specific response latencies.

### 3.2. Egocentric Digit-Based Attentional Orientation

To examine digit-induced spatial orienting from an egocentric perspective, we conducted a 2 (numerical magnitude: small [1–4] vs. large [6–9]) × 2 (probe position: left vs. right) repeated-measures ANOVA on reaction times (RTs) in the baseline condition. Results revealed no significant main effect of numerical magnitude, *F*(1, 25) = 0.224, *p* = 0.640, partial *η*^2^ = 0.009, indicating that number size alone did not influence RTs. There was a significant main effect of probe position, *F*(1, 25) = 5.458, *p* = 0.028, partial *η*^2^ = 0.179, with overall faster responses to probes on one side. Crucially, the interaction between numerical magnitude and probe position was significant, *F*(1, 25) = 21.805, *p* < 0.001, partial *η*^2^ = 0.466, indicating a robust SNARC-like effect.

**Figure 2:**
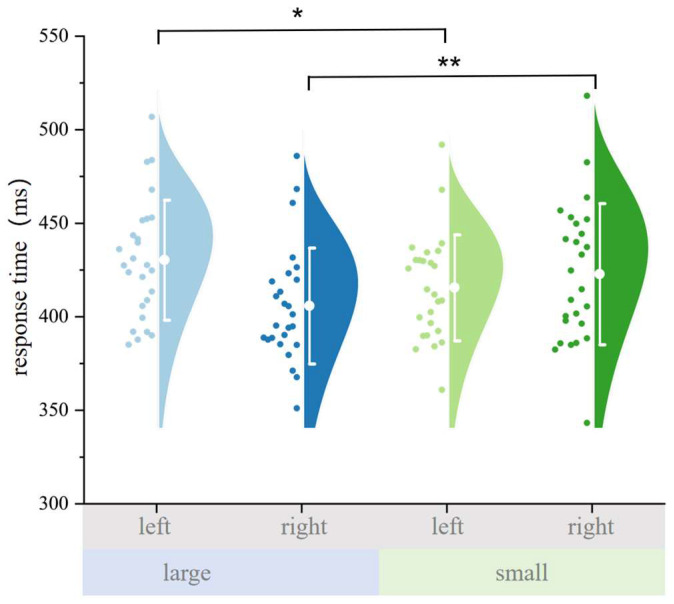
Mean reaction times (RTs) for each combination of numerical magnitude (small vs. large) and probe position (left vs. right) in the baseline (egocentric) condition. The SNARC-like interaction is evident: small digits elicited faster responses to left-side probes, whereas large digits facilitated faster responses to right-side probes. Error bars represent a ±1 standard deviation. Each dot represents a participant’s mean RT in that condition. Color coding indicates condition combinations: light blue = large number, left probe; dark blue = large number, right probe; light green = small number, left probe; dark green = small number, right probe. These results replicate the canonical egocentric SNARC pattern, whereby small numbers facilitate leftward attentional shifts, and large numbers facilitate rightward shifts, even in the absence of any spatial task demands. “*” represents *p* < 0.05; “**” represents *p* < 0.01.

Bonferroni-corrected pairwise comparisons further clarified this interaction (See Figure 2). When probes appeared on the left, responses were significantly faster for small digits compared to large digits, *t*(25) = 3.263, *p* = 0.019, MD = 14.764 ms. Conversely, when probes appeared on the right, responses were significantly faster for large digits than for small digits, *t*(25) = 3.752, *p* = 0.006, MD = 16.976 ms.

To further quantify this effect, we computed a self-consistency index, defined as the reaction time difference between inconsistent (small–right, large–left) and consistent (small–left, large–right) trials. A paired-sample *t*-test revealed that RTs were significantly slower for self-inconsistent trials compared to self-consistent trials, *t*(25) = 4.830, *p* < 0.001, MD = 16.504 ms, thus providing converging evidence for egocentric digit-induced orienting. 

No corresponding SNARC-like effect was observed along the vertical (up–down) axis in the egocentric frame. A 2 (numerical magnitude: small [1–4] vs. large [6–9]) × 2 (probe position: up vs. down) repeated-measures ANOVA revealed no significant main effect of number magnitude, *F*(1, 25) = 2.562, *p* = 0.122, partial *η*^2^ = 0.093; no significant main effect of probe position, *F*(1, 25) = 0.918, *p* = 0.347, partial *η*^2^ = 0.035; and no significant interaction, *F*(1, 25) = 0.933, *p* = 0.370, partial *η*^2^ = 0.032.

To test whether numerical extremity influenced the strength of spatial–numerical associations, we conducted a 2 (magnitude: small vs. large) × 2 (extremity: extreme vs. central) repeated-measures ANOVA on the difference scores described above. The main effect of magnitude was significant, *F*(1, 25) = 4.257, *p* = 0.049, partial *η*^2^ = 0.146, indicating that small numbers showed stronger spatial–numerical differences than large numbers. The main effect of extremity was also significant, *F*(1, 25) = 7.503, *p* = 0.011, partial *η*^2^ = 0.231, with extreme digits producing larger difference scores than central digits. The interaction between magnitude and extremity was not significant, *F*(1, 25) = 0.163, *p* = 0.690, partial *η*^2^ = 0.006. These findings suggest that both numerical polarity and extremity independently contribute to the strength of spatial–numerical mapping, without interacting.

### 3.3. Allocentric Digit-Based Attentional Orientation

In the avatar condition, we conducted a 2 (numerical magnitude: small vs. large) × 2 (probe position: up vs. down) repeated-measures ANOVA on reaction times. There was no significant main effect of numerical magnitude, *F*(1, 25) = 0.003, *p* = 0.957, partial *η*^2^ < 0.001, suggesting that number size alone did not influence response speed. The main effect of probe position was also not significant, *F*(1, 25) = 0.231, *p* = 0.635, partial *η*^2^ = 0.009. Crucially, the interaction between magnitude and probe position was significant, *F*(1, 25) = 20.589, *p* < 0.001, partial *η*^2^ = 0.452, indicating a robust SNARC-like congruency effect from the allocentric perspective.

**Figure 3:**
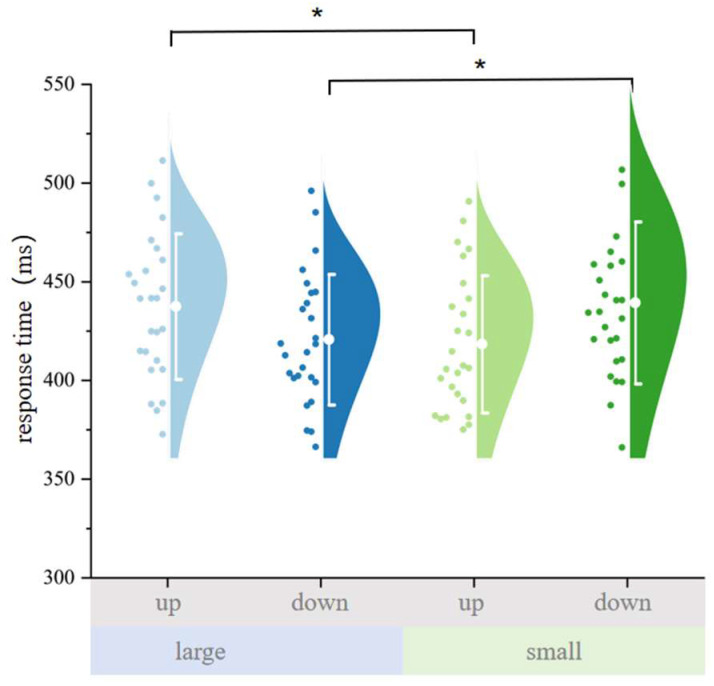
Mean reaction times (RTs) for each combination of numerical magnitude (small vs. large) and probe position (up vs. down) in the avatar (allocentric) condition. The SNARC-like interaction is evident: small digits elicited faster responses to upper probes (aligned with the avatar’s left), whereas large digits facilitated faster responses to lower probes (aligned with the avatar’s right). Error bars represent a ±1 standard deviation. Each dot represents a participant’s mean RT in that condition. Color coding indicates condition combinations: light blue = large number, up probe; dark blue = large number, down probe; light green = small number, up probe; dark green = small number, down probe. These results demonstrate an allocentric SNARC-like pattern, whereby numerical magnitude implicitly guides vertical attentional shifts consistent with the avatar’s horizontal axis. “*” represents *p* < 0.05.

Bonferroni-corrected pairwise comparisons showed that for probes presented in the upper position (above the avatar), responses to large numbers were significantly slower than to small numbers (see Figure 3), *t*(25) = 3.131, *p* = 0.026, MD = 19.069 ms. Conversely, for probes in the lower position (below the avatar), responses to large numbers were significantly faster than to small numbers, *t*(25) = 3.051, *p* = 0.032, MD = 18.613 ms. These results suggest that small numbers preferentially oriented attention upward, while large numbers oriented attention downward—consistent with an allocentric spatial–numerical mapping aligned to the avatar’s reference frame.

To test whether egocentric spatial–numerical mappings persist even when an allocentric reference frame is made salient, we performed a 2 (numerical magnitude: small vs. large) × 2 (probe position: left vs. right) repeated-measures ANOVA on trials in the avatar condition. No significant main effect of numerical magnitude was observed, *F*(1, 25) < 0.001, *p* = 0.996, partial *η*^2^ < 0.001, nor was there a main effect of probe position, *F*(1, 25) = 0.013, *p* = 0.910, partial *η*^2^ = 0.001. However, the interaction between magnitude and probe position was significant, *F*(1, 25) = 11.546, *p* = 0.002, partial *η*^2^ = 0.316, suggesting a spatial–numerical congruency effect along the egocentric horizontal axis, even in the presence of a vertical allocentric reference.

Bonferroni-corrected pairwise comparisons revealed that, although the interaction was significant, simple effects did not reach significance. For probes presented on the left, responses to large numbers were numerically slower than to small numbers, but the difference was not significant, *t*(25) = 2.296, *p* = 0.181, MD = 8.441 ms. Similarly, for probes on the right, responses to small numbers were slower than to large numbers, but again not significantly so, *t*(25) = 2.307, *p* = 0.178, MD = 8.470 ms.

**Figure 4:**
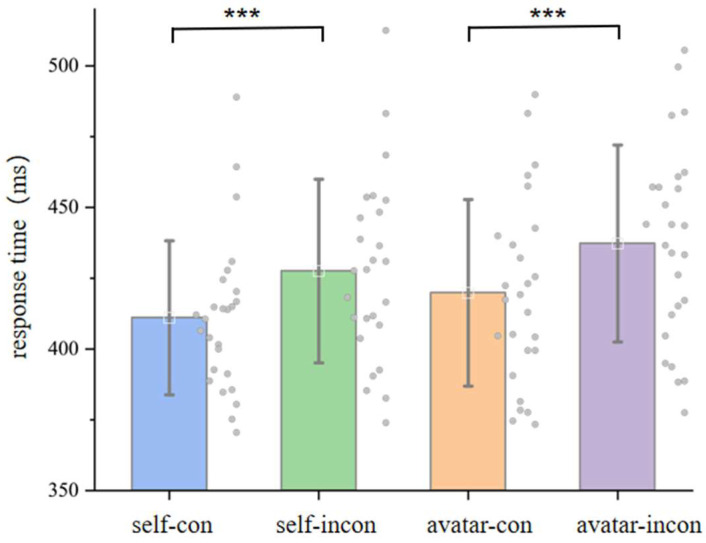
Mean reaction times (RTs) for consistent and inconsistent digit–probe pairings under egocentric (baseline) and allocentric (avatar) conditions. “Self-con” and “self-incon” refer to trials consistent and inconsistent with the canonical horizontal SNARC mapping (i.e., small–left and large–right vs. small–right and large–left), while “avatar-con” and “avatar-incon” correspond to trials aligned or misaligned with the vertical SNARC-like mapping from the avatar’s perspective (i.e., small–up and large–down vs. small–down and large–up). Error bars represent ±1 standard deviation. Each dot represents a participant’s mean RT in that condition. The results reveal significant congruency effects in both reference frames. “***” represents *p* < 0.001.

To further assess this pattern, we computed a consistency score reflecting the degree of spatial–numerical alignment from the avatar’s perspective. Specifically, trials were classified as consistent if small numbers were paired with upper probes and large numbers with lower probes and as inconsistent if the pairings were reversed. A paired-sample t-test showed that response times were significantly slower for inconsistent pairings than for consistent ones, *t*(25) = 4.157, *p* < 0.001, MD = 17.403 ms, providing converging evidence for allocentric digit-induced attentional orienting (see Figure 4).

Finally, we examined whether numerical extremity modulated digit-induced spatial biases in the avatar condition, using the same 2 (numerical magnitude: small vs. large) × 2 (extremity: extreme vs. central) repeated-measures ANOVA approach as in the egocentric condition. Consistent with earlier results, difference scores were calculated separately for small and large digits based on the expected spatial alignments and then compared across extremity levels.

The main effect of numerical magnitude was not significant, *F*(1, 25) = 1.635, *p* = 0.213, partial *η*^2^ = 0.061, indicating no reliable difference in spatial association strength between small and large numbers. The main effect of extremity also failed to reach significance, *F*(1, 25) = 3.081, *p* = 0.091, partial *η*^2^ = 0.110, although descriptively, extreme digits tended to elicit slightly stronger congruency effects than central digits. The interaction between magnitude and extremity was likewise non-significant, *F*(1, 25) = 1.737, *p* = 0.200, partial *η*^2^ = 0.065. Together, these results suggest that—unlike in the egocentric condition—numerical extremity did not substantially modulate avatar-based spatial–numerical mapping.

## 4. Discussion

The present study provides compelling evidence that spatial–numerical associations —long assumed to reflect culturally internalized and egocentrically grounded mappings (Shaki & Fischer, 2018)—can be flexibly reconfigured based on another person’s reference frame. By employing a digit-primed visual detection paradigm, we show that the presence of a task-irrelevant avatar induces a robust SNARC-like effect along the avatar-defined vertical axis, indicating that digit-induced attentional biases can realign to an allocentric reference frame without explicit instruction or strategic incentive.

### 4.1. Egocentric SNARC-like Effect and Its Spatial Characteristics

In the baseline condition, where no avatar was present, we replicated the classic SNARC pattern along the horizontal axis: small numbers facilitated the faster detection of left-sided probes, whereas large numbers facilitated right-sided probes’ detection. This finding reinforces the notion that number magnitude can automatically bias visuospatial attention along culturally dominant axes (Moorkens et al., 2025; Roth et al., 2025). Importantly, this horizontal SNARC was absent along the vertical axis in the egocentric frame, confirming prior work suggesting that the horizontal plane holds a privileged status in number–space associations due to reading/writing direction and embodied motor experience (Shaki et al., 2009; Gevers et al., 2006).

Moreover, we found that numerical extremity significantly modulated the strength of the SNARC-like effect in the egocentric condition: extreme digits (1/2 and 8/9) elicited stronger congruency effects than central digits (3/4 and 6/7), consistent with theories of polarity correspondence (Proctor & Cho, 2006) and magnitude salience (Schwarz & Keus, 2004). These results affirm that not all digits are equally potent in driving spatial codes, and that extremity may amplify the alignment between magnitude and spatial attention.

### 4.2. Avatar-Induced Allocentric Mapping: Evidence for Embodied Perspective Taking

Strikingly, in the avatar condition, SNARC-like effects emerged along the vertical axis defined by the avatar’s body orientation—despite the avatar being task-irrelevant, and no vertical bias being evident in the baseline condition. Small numbers facilitated attention toward the avatar’s “upper” space, while large numbers oriented attention “downward”, suggesting a re-anchoring of numerical–space mapping to the other’s visuospatial reference frame. This finding supports the idea that visual perspective taking can occur spontaneously and implicitly (Samson et al., 2010; Surtees et al., 2013), even in the absence of explicit task demands.

The emergence of such allocentric effects demonstrates that spatial–numerical mappings are not solely the product of internal symbolic structures but are sensitive to external social cues. Prior research has shown that the mere presence of another person—regardless of task relevance—can influence attentional allocation, suggesting that social information is spontaneously integrated into cognitive processing (Furlanetto et al., 2016; Bukowski et al., 2015). Our results extend this line of work by showing that the spatial orientation of another agent can reshape even abstract cognitive representations, such as number–space associations. Specifically, the observed realignment of attentional biases along the avatar’s vertical axis implies that participants are engaged in an embodied simulation of the other’s spatial perspective. This supports embodied theories of numerical cognition, which argue that abstract magnitude processing is grounded in sensorimotor experience and dynamically responsive to bodily and interpersonal context (Lakoff & Núñez, 2000; Fischer & Coello, 2016).

### 4.3. Reference Frame Flexibility and Socially Contingent Embodiment

Crucially, the original horizontal SNARC effect remained statistically reliable even in the presence of the avatar, indicating that egocentric spatial coding was still co-activated. However, the interaction between number magnitude and horizontal probe location was notably weakened in the avatar condition. This partial suppression suggests a resource-based competition between spatial codes (Avraamides et al., 2004; R. F. Wang & Spelke, 2002), in which the allocentric frame, bolstered by the avatar’s embodied salience, modulated attentional prioritization without fully overriding the egocentric default.

These results challenge static models of spatial–numerical mapping and point instead to a dynamic, multi-referential system in which spatial codes are continuously shaped by both internal and external factors. Rather than relying on a single dominant schema—either egocentric or allocentric—our findings support a view in which multiple reference frames can be co-activated and flexibly reweighted based on contextual and embodied cues (Avraamides et al., 2004; Guo et al., 2024).

Further supporting this view, we observed a dissociation in extremity-based modulation. While egocentric SNARC effects were significantly amplified for extreme digits—consistent with prior research on polarity and spatial competition (de Hevia et al., 2008)—this modulation vanished under allocentric mapping. In the vertical SNARC-like effect, digit extremity had no discernible impact, suggesting a qualitative difference in processing mechanisms. We propose that egocentric mappings remain sensitive to internal symbolic structures such as magnitude polarity and extremity, whereas allocentric mappings—especially those grounded in embodied simulation of another’s perspective—are driven more by external spatial anchoring and less by semantic features.

Together, these findings advance a multi-referential framework of number–space associations, in which egocentric and allocentric codes can coexist and compete, with relative dominance shaped by tasks’ context, social embodiment, and semantic load (Avraamides et al., 2004; Surtees et al., 2013). This dynamic interplay illustrates how abstract cognitive structures are not isolated from social context but instead deeply interwoven with embodied social cognition.

### 4.4. Theoretical and Methodological Contributions

The present study offers several key contributions to the literature on numerical cognition and social attention. First, by employing a digit-primed detection paradigm rather than traditional classification or parity tasks, we isolate early attentional orienting from later motor or decision-related processes, providing more direct evidence that spatial–numerical associations can arise at the perceptual level. Second, our findings extend research on implicit visual perspective taking (VPT) by demonstrating that such embodied processes can reshape even highly abstract representations such as number magnitude. Third, the observed dissociation between egocentric and allocentric SNARC-like effects reveals that numerical–spatial mappings are not fixed to the bodily self but can flexibly reorganize across reference frames, highlighting the interplay between ego- and allocentric representations in shared cognitive space.

### 4.5. Limitations and Future Directions

Despite these contributions, several limitations of the current design warrant caution and suggest directions for future research. Most notably, the avatar was consistently presented on the participant’s left side throughout the experiment. While the allocentric SNARC-like effect we observed emerged along the vertical axis—an axis where no egocentric SNARC effect was found in the baseline condition—it remains possible that the fixed lateral placement of the avatar introduced unintended spatial asymmetries or attentional biases. In other words, the remapping of number–space associations into the avatar-defined frame may have been partially confounded with a general leftward attentional bias toward the avatar’s physical location. To disentangle true reference frame adoption from spatial proximity effects, future studies should counterbalance the avatar’s location across both left and right visual fields.

Second, although the current study provides initial evidence that spatial–numerical associations can be reorganized relative to a task-irrelevant agent’s viewpoint, it remains unclear whether such remapping is modulated by the agent’s socially attributed characteristics. In our design, the avatar lacked intentionality, emotional expressivity, or social affiliation cues, which are known to influence how deeply others’ perspectives are simulated. For instance, Simpson and Todd (2017) showed that participants experienced greater egocentric interference when adopting the viewpoint of an ingroup rather than outgroup member, suggesting that shared group identity enhances the salience of the other’s perspective. Similarly, the altercentric cognition framework (Kampis & Southgate, 2020) highlights that human information processing is inherently shaped by the perceived mental states and attentional focus of others—even when those others are not directly relevant to the task. Moreover, recent work on human–avatar interaction suggests that experiencing a sense of embodiment and control over an avatar can enhance spontaneous perspective taking and lead to the merging of avatar–self (Böffel & Müsseler, 2019; Müsseler et al., 2022).

Future work should systematically manipulate social cues—such as perceived intentionality, emotional expressivity, and group affiliation—to clarify how these attributes modulate the strength, directionality, and automaticity of reference frame realignment. Such variables are not merely peripheral; they fundamentally shape the extent to which others’ perspectives are embodied and integrated into spatial processing. This line of inquiry would help determine whether the allocentric remapping observed here reflects a general sensitivity to social presence or a more selective process gated by interpersonal relevance and cognitive resonance. Clarifying these boundary conditions will be essential for advancing a socially grounded account of embodied spatial cognition.

## 5. Conclusions

In summary, the current findings demonstrate that SNARC-like effects are not fixed products of internal symbolic representation but can be flexibly reorganized in accordance with the perceived spatial reference frame of another agent. This reveals the profound permeability of numerical cognition to social and embodied factors, even at early stages of attentional orienting. Far from being an isolated symbolic domain, number processing is deeply integrated with the bodily, spatial, and social systems that support adaptive interaction with the world. Critically, the spontaneous re-anchoring of spatial–numerical mappings to another’s viewpoint not only illustrates the embodied nature of magnitude processing but also underscores the role of perceptual simulation in fostering intuitive social understanding—allowing individuals to align their attention with others’ perspectives in a fast and automatic manner.

## Figures and Tables

**Table 1:** Conditions of consistent and inconsistent trials in egocentric and allocentric reference frames.

Trial Types	Numerical Magnitude	Probe Location
Self-consistent	Small	Left
	Large	Right
Self-inconsistent	Small	Right
	Large	Left
Avatar-consistent	Small	Up
	Large	Down
Avatar-inconsistent	Small	Down
	Large	Up

## Data Availability

The original data presented in this study are openly available.
